# Effects on secondary outcomes following a three-month personalized app-based lifestyle intervention among working adults: a three-armed randomized controlled trial

**DOI:** 10.1038/s41598-026-54919-w

**Published:** 2026-05-31

**Authors:** Helén Eke, Daniel Söderberg, Linnea Sjöblom, Stephanie E Bonn, Ylva Trolle Lagerros

**Affiliations:** Department of Medicine, Huddinge, Karolinska Institutet, H7/Neo/Helén Eke, Huddinge, 141 83 Stockholm Sweden

**Keywords:** Diet, Health promotion, mHealth, Physical activity, Sleep, Stress, Health care, Medical research, Psychology, Psychology, Risk factors

## Abstract

Lifestyle improvements can bring individual and societal benefits. Most interventions are standardized; however, personalized interventions may be more effective. We examined the effect of a personalized app‑based lifestyle intervention, with or without monthly coaching, on physical activity, diet, alcohol, sleep, stress, and smoking, in a three‑month randomized controlled trial. Office workers and bus drivers (*n* = 209) were randomized 1:1:1 to app, app-coach, or control group. Differences in change from baseline to follow‑up were analysed using robust linear regression, with the control group as the reference. Participants were on average 48.2 years and 42% were female. Both intervention groups increased minutes of exercise (app group β = 15.7 min/week, 95% CI: 3.5, 27.9; app-coach group β = 13.8 min/week, 95% CI: 1.4, 26.1). Compared to the control group, participants in the app-coach group increased their vegetable intake (β = 51.8 g/day, 95% CI: 2.5, 101.1) and fruit and vegetable intake (β = 87.7 g/day 95% CI: 8.9, 166.6). Participants in the app-coach group also increased time in bed (β = 0.43 h/night, 95% CI: 0.05, 0.81) and sleep duration (β = 0.40 h/night, 95% CI: 0.01, 0.78). No other outcomes differed between groups. While the effects seen on physical activity, diet and sleep were limited, even small behavioural changes can benefit health. Though, the effect of coaching needs further evaluation. Future personalized app-based interventions have potential to improve lifestyle behaviours.

**Supplementary Information:**

The online version contains supplementary material available at 10.1038/s41598-026-54919-w.

## Introduction

Noncommunicable diseases such as type 2 diabetes, cancer, and cardiovascular disease, contribute to 70% of annual deaths worldwide^1^. The majority of the modifiable risk factors for these diseases are lifestyle-related, including physical activity, diet, alcohol, sleep, stress and smoking^2–4^. These lifestyle behaviours are often related with each other^5–11^. For example, a healthy diet has been shown to be associated with sufficient levels of physical activity^8^. Furthermore, higher levels of stress have been shown to be associated with a lower sleep quality^9^. These associations are often bidirectional; a healthy dietary pattern has been associated with better sleep quality^11^, while short sleep duration was shown to be associated with an increased energy intake^10^. In addition, improvements in one lifestyle behaviour are often associated with improvements in other behaviours, i.e. transfer effects are common^5–7^. For example, improvements in physical activity have been shown to be associated with improvements in diet and sleep, decreased stress levels and lower alcohol consumption^5^. Improvements in these lifestyle behaviours can imply large benefits on both individual and societal level.

A wide range of lifestyle behaviours, for example physical activity, diet, and sleep, as well as self-management of several diseases, can be successfully targeted through mobile health (mHealth) interventions^12–15^. mHealth interventions offer great accessibility and are cost-effective^16,17^. However, while individuals may have different needs and preferences, most interventions are standardized. In standardized interventions, user-engagement and adherence, which are key factors for effectiveness of an intervention, can be difficult to maintain^18–21^. Instead, personalized interventions target behaviours that are of specific interest to the individual. Such interventions have the potential to increase engagement and sustained adherence to the intervention, which may in turn improve overall health more effectively^18^. Another way to enhance the effectiveness of mHealth interventions is by including a coach, which has been shown to, for example, increase weight loss^22^ and improve behavioural change and management in persons with type 2 diabetes^23,24^.

The workplace setting has been identified as an ideal environment for health interventions, offering significant benefits for health and wellbeing, while also enhancing productivity and job satisfaction^25,26^. In an umbrella review, Virtanen et al.^27^ concluded that workplace interventions can be effective to improve for example physical activity, diet and stress. Similarly, a systematic review by Sevic et al.^28^ examined workplace-based eHealth interventions. They reported small to large effects, especially for physical activity, although several studies also showed non-significant results. While highlighting the potential of these interventions, concerns were raised regarding the quality of the studies and authors expressed a need for more robust evidence on the effectiveness.

Recently, Seiterö et al.^29^ included more than 700 high school students in a 16-week mHealth intervention targeting multiple lifestyle behaviours. Following baseline screening, the intervention was automatically personalized to address behaviours needing improvement and continuously adapted based on each participant’s performance. A significant increase in fruit and vegetable consumption was found in the intervention group, along with indications of improved physical activity levels and reduced rates of heavy drinking. Moreover, Mateos et al.^30^ included patients with type 2 diabetes who started treatment with a Glukagon-Like Peptide-1 analogue. Half of the patients were randomized to receive additional digital coaching support. Both groups decreased their body weight and improved their glycated haemoglobin (HbA1c), but the improvements were larger in the group that also received the digital coaching. Further, Andargeery et al.^31^ performed an randomized controlled trial (RCT) that compared the delivery-mode of a multi-dimensional health intervention. They found similar improvements in the mHealth intervention group compared to the face-to-face group and concluded that the mHealth-delivery improved the cost-effectiveness of the intervention.

Although conducted in different populations and contexts, previous studies consistently demonstrate the potential of mHealth interventions to improve lifestyle behaviours and clinical outcomes. Despite this, studies examining personalized lifestyle interventions in workplace settings are scarce. At the same time, the World Health Organization has stated that there is now an expanded employer responsibility to promote not only occupational safety, but also lifestyle‑related behaviours and prevention of noncommunicable diseases^25^. This combination of limited evidence and expanded responsibility highlights the need for further evaluations of workplace-based personalized interventions. To support working adults in their workplace setting, the personalized lifestyle intervention *the Health Integrator* was developed as part of the European collaboration *The health movement* – *for patient empowerment*^32^. The Health Integrator was developed through an intensive five‑day design sprint involving experts from science, business, and innovation, and then further refined in collaboration with relevant stakeholders prior to launch. The intervention has previously been shown to improve clinical outcomes of for example waist circumference and Body Mass index (BMI)^33^.

In this study, we aimed to assess the effect of this personalized app-based lifestyle intervention, with or without additional monthly coach support, on secondary outcomes of physical activity, diet, alcohol, sleep, stress and smoking, among working adults in a three-month RCT. We hypothesized that study participants in the intervention groups would improve their lifestyle behaviours compared to participants in the control group.

## Results

**Fig. 1:**
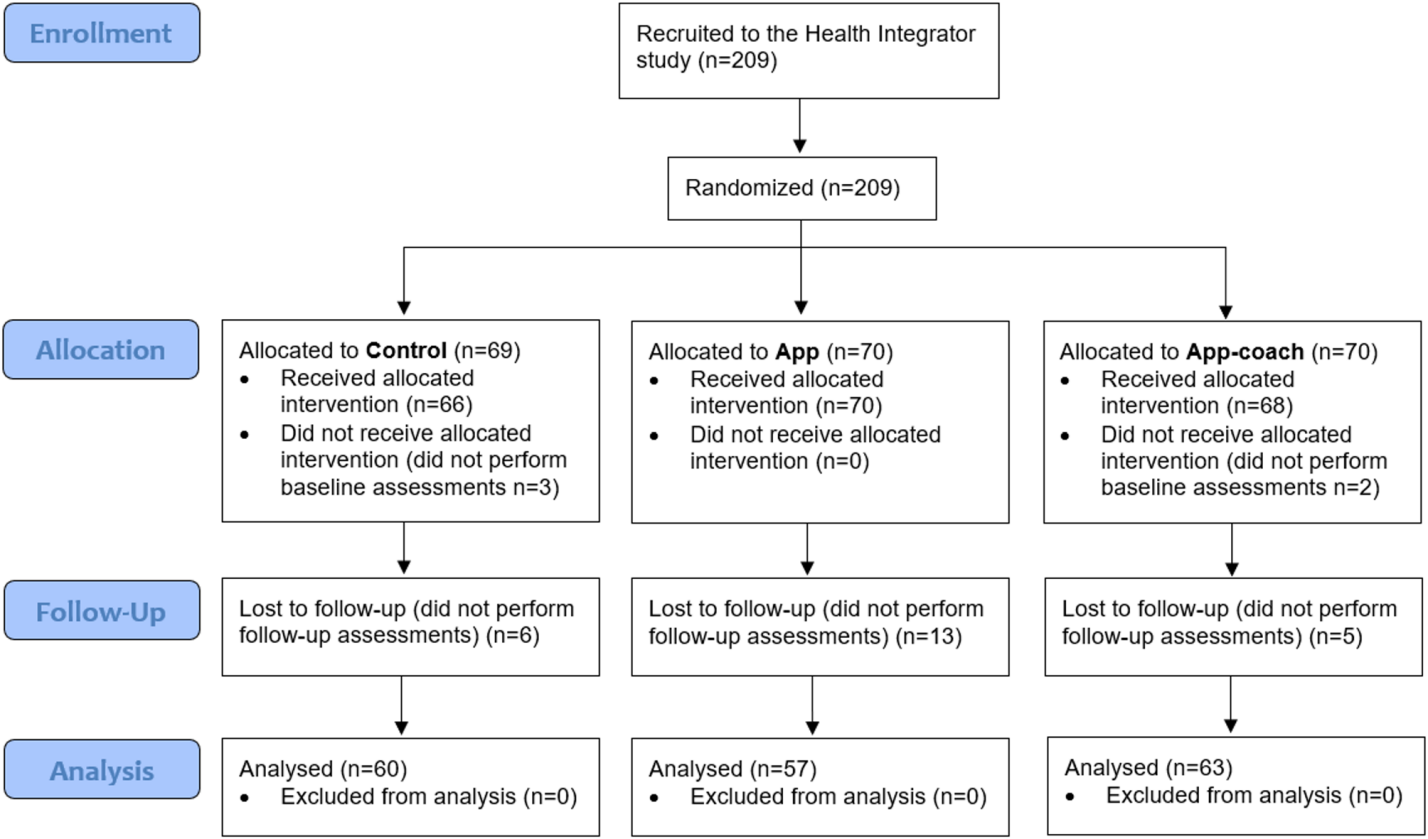
CONSORT 2010 flow diagram of the Health Integrator study, a three-month randomized controlled trial, performed among working adults in Sweden. All participants who responded to the research questionnaire at baseline and follow-up were included in the analyses (*n* = 180).

In total, 209 participants were included and randomized in the Health Integrator study. To analyse the intervention effect, we included those who responded to the research questionnaire at baseline and follow-up (*n* = 180), Fig. 1. Table 1 shows baseline characteristics of participants included in further analyses. Participants were on average 48.2 years old and 42% (*n* = 75/180) were female. Almost half, 46% (*n* = 83/180), were bus drivers. No statistically significant differences in any characteristics were seen between the three intervention groups. Baseline characteristics of all 204 participants with baseline data are presented in Supplementary Table S1 online.

**Table 1:** Baseline characteristics of participants included in analyses in the Health Integrator study, a three-month randomized controlled trial, performed among working adults in Sweden.

	All (n = 180) mean (SD)	Control (n = 60) mean (SD)	App (n = 57) mean (SD)	App-coach (n = 63) mean (SD)	*p * ^a^
Age, years	48.2 (10.2)	48.7 (8.8)	47.3 (11.0)	48.6 (10.8)	.73
BMI, kg/m^2^	27.0 (4.6)	26.9 (4.1)	26.7 (5.0)	27.4 (4.8)	.73
Physical activity, min/week					
Everyday activity	187.6 (103.7)	172.3 (107.8)	204.5 (95.2)	186.9 (106.3)	.24
Exercise	59.3 (47.5)	65.0 (48.3)	53.7 (49.5)	58.8 (44.9)	.44
Total physical activity	246.8 (132.6)	237.3 (143.5)	258.2 (122.6)	245.7 (131.7)	.70
Diet, intake/day ^b^					
Energy, kcal	2138 (872)	2262 (1124)	2026 (715)	2118 (711)	.35
Fruit, g	204.6 (184.3)	207.5 (227.5)	190.0 (152.5)	215.2 (165.9)	.75
Vegetables, g	164.3 (130.7)	151.4 (165.1)	155.1 (94.2)	185.0 (121.1)	.30
Fruit and vegetables, g	369.0 (278.2)	358.8 (366.9)	345.0 (199.6)	400.2 (240.2)	.53
Alcohol, units/day ^b^	0.8 (1.1)	1.0 (1.3)	0.8 (0.9)	0.6 (0.9)	.17
Sleep, h/night					
Time in bed	7.1 (1.3)	7.0 (1.4)	7.3 (1.2)	7.2 (1.2)	.38
Sleep duration	6.8 (1.3)	6.6 (1.5)	6.9 (1.3)	6.9 (1.2)	.41
Stress, score ^c^	22.8 (7.7)	23.4 (8.5)	22.4 (7.5)	22.5 (7.1)	.73
	n (%)	n (%)	n (%)	n (%)	*p* ^d^
Sex					.91
Females	75 (41.7)	24 (40.0)	25 (43.9)	26 (41.3)	
Males	105 (58.3)	36 (60.0)	32 (56.1)	37 (58.7)	
Education level					.97
≤ 12 years	85 (47.2)	29 (48.3)	27 (47.4)	29 (46.0)	
> 12 years	95 (52.8)	31 (51.7)	30 (52.6)	34 (54.0)	
Type of work					.52
Office employee	97 (53.9)	32 (53.3)	34 (59.6)	31 (49.2)	
Bus driver	83 (46.1)	28 (46.7)	23 (40.4)	32 (50.8)	
Smoking status					.26
Smoker	13 (7.2)	7 (11.7)	3 (5.3)	3 (4.8)	
Non-smoker	167 (92.8)	53 (88.3)	54 (94.7)	60 (95.2)	
Physical activity, ≥ 150 min/week					.52
Yes	137 (76.1)	43 (71.7)	46 (80.7)	48 (76.2)	
No	43 (23.9)	17 (28.3)	11 (19.3)	15 (23.8)	
Target behaviour ^e^					
Physical activity	80 (66.7)	–	37 (64.9)	43 (68.3)	.70
Diet	65 (54.2)	–	34 (59.6)	31 (49.2)	.25
Alcohol	0 (0.0)	–	0 (0.0)	0 (0.0)	-
Sleep	38 (31.7)	–	17 (29.8)	21 (33.3)	.68
Stress	51 (42.5)	–	22 (38.6)	29 (46.0)	.41
Smoking	2 (1.7)	–	2 (3.5)	0 (0.0)	.13

BMI = Body Mass Index, kcal = kilocalorie, ^a ^analysis of variance, ^b ^92-item Food Frequency Questionnaire, ^c ^Perceived Stress Scale, ^d ^chi-squared tests, ^e ^possible to target one or more behaviours, only available in the intervention groups (n = 120).

Participants in the two intervention groups chose to target one or more lifestyle behaviours. The most targeted behaviour in both intervention groups was physical activity (app group *n* = 37/57, 64.9%; app-coach group *n* = 43/63, 68.3%), followed by diet (app group *n* = 34/57, 59.6%; app-coach group *n* = 31/63, 49.2%). Stress (app group *n* = 22/57, 38.6%; app-coach group *n* = 29/63, 46.0%) and sleep (app group *n* = 17/57, 29.8%; app-coach group *n* = 21/63, 33.3%) were also commonly targeted, while only two participants targeted smoking and no participant targeted alcohol. There was no statistically significant difference between the intervention groups in terms of the distribution of participants that targeted any of the lifestyle behaviours (all *p*≥.13), Table 1.

The number of targeted behaviours by a participant ranged from none to four. In the app group, it was most common to target two behaviours (*n* = 22/57, 38.6%) while in the app-coach group, it was most common to target one behaviour (*n* = 21/63, 33.3%). The difference in the distribution of number of targeted behaviours between the groups was not statistically significant (*p*=.65). For those that targeted more than one behaviour, the most common combinations were similar in both intervention groups, i.e. to combine physical activity with either diet, sleep or stress, or to combine sleep with stress.

Among the participants randomized to the app-coach group, all but two participants took part in the first coaching session after one month (*n* = 61/63, 96.8%). Almost all participants also completed the second session after two months (*n* = 61/63, 96.8%).

### Intervention effect

The results of the intervention effect are presented in Table 2. Results of outcome assessments at baseline and follow-up are presented in Supplementary Table S2 online. Participants in both intervention groups had increased their weekly exercise more than the control group at the three-month follow-up (app group β = 15.7 min/week, 95% CI: 3.5, 27.9; app-coach group β = 13.8 min/week, 95% CI: 1.4, 26.1). With the control group as the reference, an increased vegetable intake (β = 51.8 g/day, 95% CI: 2.5, 101.1) and fruit and vegetable intake (β = 87.7 g/day, 95% CI: 8.9, 166.6) were seen in the app-coach group. This was not seen in the app group. Time in bed increased significantly in the app-coach group compared to the control group (β = 0.43 h/night, 95% CI: 0.05, 0.81). Sleep duration increased as well and was statistically significant compared to the control group (β = 0.40 h/night, 95% CI: 0.01, 0.78). We did not find any other statistically significant intervention effects for physical activity, diet, alcohol, sleep, or stress.

**Table 2:** Robust linear regression examining the difference in change between baseline and follow-up, in comparison to the control group.

	Group	*n*	Difference ^a^	Linear regression model ^b^
			mean (SD)	β (95% CI)
Everyday activity, min/week ^c^	Control	60	0.3 (71.9)	ref
	App	57	–11.6 (93.9)	1.8 (–25.7, 29.3)
	App-coach	63	–0.5 (106.9)	5.5 (–24.0, 35.0)
Exercise, min/week ^c^	Control	60	–5.0 (41.7)	ref
	App	57	15.5 (39.6)	**15.7 (3.5**,** 27.9)**
	App-coach	63	11.4 (37.2)	**13.8 (1.4**,** 26.1)**
Total physical activity, min/week ^c^	Control	60	–4.8 (89.2)	ref
	App	57	3.9 (107.8)	16.9 (–15.6, 49.3)
	App-coach	63	11.0 (124.3)	19.0 (–15.5, 53.5)
Energy intake, kcal/day ^d^	Control	59	–186 (1011)	ref
	App	55	64 (785)	104 (–149, 357)
	App-coach	63	–21 (765)	83 (–153, 318)
Fruit, g/day ^d^	Control	59	–20.9 (196.3)	ref
	App	55	2.1 (130.7)	12.9 (–31.1, 56.9)
	App-coach	63	7.8 (183.2)	32.4 (–12.7, 77.5)
Vegetables, g/day ^d^	Control	59	–13.8 (166.2)	ref
	App	55	8.5 (85.8)	25.3 (–10.4, 60.9)
	App-coach	63	19.7 (130.2)	**51.8 (2.5**,** 101.1)**
Fruit and vegetables, g/day ^d^	Control	59	–34.8 (344.1)	ref
	App	55	10.6 (160.1)	38.9 (–27.9, 105.6)
	App-coach	63	27.5 (260.2)	**87.7 (8.9**,** 166.6)**
Alcohol, units/day ^d^	Control	60	–0.2 (0.6)	ref
	App	55	–0.0 (0.7)	0.1 (–0.1, 0.3)
	App-coach	63	–0.1 (0.7)	–0.0 (–0.3, 0.2)
Time in bed, h/night ^c^	Control	59	0.02 (1.25)	ref
	App	56	0.10 (1.31)	0.25 (–0.11, 0.62)
	App-coach	57	0.25 (1.25)	**0.43 (0.05**,** 0.81)**
Sleep duration, h/night ^c^	Control	59	0.08 (1.27)	ref
	App	56	–0.04 (1.53)	0.06 (–0.35, 0.47)
	App-coach	57	0.30 (1.27)	**0.40 (0.01**,** 0.78)**
Stress, score ^e^	Control	60	–1.9 (6.0)	ref
	App	55	–2.7 (5.1)	–1.1 (–3.0, 0.9)
	App-coach	63	–2.4 (6.2)	–0.8 (–2.8, 1.2)

Bold numbers imply statistical significance. Outcomes in the Health Integrator study, a three-month randomized controlled trial, performed among working adults in Sweden.

kcal= kilocalorie, ^a^ difference baseline and follow-up, ^b^ adjusted for baseline value, ^c^ self-reported, ^d^ 92-item Food Frequency Questionnaire, ^e^ Perceived Stress Scale.

The effects seen in main analyses remained in the sensitivity analyses using the last observation carried forward method for exercise (app group β = 14.4 min/week, 95% CI: 3.4, 25.4; app-coach group β = 12.8 min/week, 95% CI: 1.2, 24.3), vegetable intake (app-coach group β = 47.1 g/day, 95% CI: 1.1, 93.2) and fruit and vegetable intake (app-coach group β = 81.1 g/day, 95% CI: 5.9, 156.4). For time in bed and sleep duration, the effects did not remain statistically significant (app-coach group: time in bed β = 0.28 h/night, 95% CI: -0.06, 0.63; sleep duration β = 0.27 h/night, 95% CI: -0.07, 0.62). In the second sensitivity analysis, we instead used multiple imputations to impute the missing outcome variables at follow-up. For exercise, the estimates remained statistically significant in the app group (β = 13.3 min/week, 95% CI: 0.9, 25.8), but not in the app-coach group (β = 11.6 min/week, 95% CI: -0.8, 24.0). Further, neither the effect on vegetable intake (app-coach group β = 46.9 g/day, 95% CI: -1.3, 95.1) nor the effect on fruit and vegetable intake combined (app-coach group β = 77.7 g/day, 95% CI: -2.9, 158.2) remained significant. Time in bed remained statistically significant (app-coach group β = 0.40 h/night, 95% CI: 0.01, 0.79), but sleep duration did not (app-coach group β = 0.40 h/night, 95% CI: -0.01, 0.81).

### Stratified analyses

Results stratified by targeted behaviour are found online in Supplementary Table S2 (baseline and follow-up assessments) and Supplementary Table S3 (intervention effect and sample sizes). Results for weekly exercise remained significant for the participants who targeted physical activity (app group β = 21.6 min/week, 95% CI: 8.1, 35.0; app-coach group β = 16.2 min/week, 95% CI: 2.8, 29.5), compared to the control group. The effects seen in the app-coach group on vegetable intake, and fruit and vegetable intake combined, did not remain statistically significant in the stratified analyses. The increased duration of time in bed and sleep observed in the app-coach group compared to the control group did not remain statistically significant in the stratified analysis. Instead, significant effects were seen among participants who targeted their sleep in the app group, for time in bed (β = 0.48 h/night, 95% CI: 0.08, 0.87) and sleep duration (β = 0.51 h/night, 95% CI: 0.12, 0.89). No statistically significant effects were seen among those who did not target physical activity, diet, or sleep.

## Discussion

In this RCT, we assessed the effect of a personalized app-based lifestyle intervention together with an initial health coach session, with or without additional monthly coach support, on lifestyle behaviours among working adults. Overall, the intervention had limited effects after three months. However, we found a positive intervention effect on weekly exercise in both intervention groups. We also found that the intervention may increase fruit and vegetable intake, time in bed and sleep duration. We did not see any intervention effects on alcohol intake, stress or smoking.

As the improvements we observed were limited, the clinical relevance of our findings can be discussed. The modest increase of fifteen min/week of exercise is in line with previous physical activity interventions^34^. However, even small improvements beyond recommended levels can offer additional health benefits^35^. Regarding fruit and vegetable intake, a small decrease in fruit and vegetable intake was seen in the control group, whereas a modest increase was seen in the app-coach group, resulting in a positive intervention effect. This may be of clinical relevance, as each daily serving of fruit and vegetables has been associated with a 5% decrease of all-cause mortality, especially among individuals not reaching the recommended intake^36^. The increase seen in sleep duration, approximately 0.4 h/night, may also bring health benefits since the mean sleep duration at baseline was slightly below the recommended 7–9 h/night duration^2^. All steps towards an adequate sleep duration have shown a dose-response relationship with decreased risk of several non-communicable diseases^37^. Overall, these results remained in sensitivity analysis. However, some estimates were now borderline significant, which reflect the limited sample size, as analyses address secondary outcomes, while the trial was powered for the primary outcome. As such, we believe that the small improvements seen in our study can still be of clinical relevance.

There are several possible explanations to our limited findings. Overall, our study population may have been healthier than the general population already at baseline. This is supported by more people reaching the recommendation for physical activity (76% in the study sample compared to 66% in the general population^35)^. They also had a higher daily fruit and vegetable intake of approximately 70 g more compared to the general population^38^. It may therefore have been more difficult to further improve behaviours already considered good. In addition, due to the personalized design, we did not expect all participants to improve in all behaviours. Since each participant focused on different behavioural goals based on their needs and preferences, improvements on individual level were expected to vary both in terms of type of behaviour and extent of improvement.

Given the limited intervention effects overall, there is not enough evidence to draw conclusions on the additional coaching specifically. Although the app‑coach group showed statistically significant improvements in sleep outcomes and vegetable intake, estimates in the app group were in the same direction. Considering the limited sample size and the personalized design, the effect of coaching requires further evaluation. Previous studies have found that health-coaching led to improvements of the targeted lifestyle behaviours^39,40^, which could not be confirmed in our study. Further, Zarski et al.^41^ pooled data from several RCTs and found that coaching was associated with better adherence, which in turn has been linked to lower dropout rates^42^. However, we observed low dropout rates across all groups and not only in the group that received additional monthly coaching.

Our results should be interpreted in relation to similar studies. Viester et al.^43^ also performed a lifestyle intervention in a workplace setting, with the aim to improve physical activity and diet among male construction workers. The intervention group received individually tailored interventions based on a baseline screening and a face-to-face health coaching session^43^. After six months, they had increased their physical activity and decreased their intake of sugar-sweetened beverages, in comparison to the control group. Their study has similarities with ours in terms of the baseline screening and individual health coaching, but their aim to improve physical activity and diet was set for all participants. Even though our participants could decide the focus of the intervention themselves and had the opportunity to choose among several interventions relating to six different lifestyle behaviours, many participants chose the combination of physical activity and diet. We also found improvements in physical activity, which indicates that both standardized interventions with predefined goals, as well as personalized interventions with individual-specific goals, can bring positive effects.

Schoeppe et al.^44^ concluded in a systematic review that multi-component interventions seem to be more effective than stand-alone interventions. While transfer effects is common, participants in our study were able to choose interventions within all the behaviours they wanted to target, limiting our ability analyse these effects separately. In our analyses stratified by targeted behaviour, the results for physical activity and sleep could be interpreted in such a way that the behaviour must be targeted to create a change, as effect estimates only were significant in groups that had targeted the behaviour. However, due to small sample sizes, our stratified analyses must be interpreted as exploratory and with caution. Future studies would benefit from including larger sample sizes to more thoroughly assess each target behaviour.

Our population was similar to the general population regarding smoking habits and alcohol intake^38,45^. Although the majority of smokers in Sweden express a willingness to quit^46^, few smokers (*n* = 2/13) targeted smoking cessation in our study. Further, participants consumed on average 0.8 standard alcohol units per day, and while reducing alcohol intake is beneficial for all individuals who consume alcohol^47^, no participant in our study targeted alcohol intake. Common barriers to engage in workplace interventions targeting these behaviours include a low perceived need for external support^48^ and low readiness to change^49^. Despite these challenges, the workplace remains a promising setting for behaviour change, offering access to individuals who may not seek help elsewhere and utilizing the large amount of time that individuals spend at work^50^.

This study has some weaknesses. Herein, we analysed secondary outcomes, and the power-calculation was based on the primary outcome health-related quality of life^51^. Our initial aim was to also examine changes in smoking, but we could not do that due to few smokers at baseline. In addition, we could not perform stratified analyses on alcohol, since no participant targeted alcohol intake. Further, our outcomes are based on self-reported data, which comes with uncertainties such as recall and social desirability bias. Participants may over- or underestimate their self-reported health behaviours, which could influence the accuracy of our findings. This potential bias should be considered when interpreting the results, especially since we were unable to blind the participants to their group allocation. Another limitation is the absence of fidelity data, as adherence data to the intervention services was unavailable in the intervention groups. The high participation rates seen in the app-coach group for the coaching sessions does not necessarily reflect engagement in target behaviours. Our results regarding sleep are limited by the use of a single-night assessment, rather than habitual sleep patterns. Assessments for multiple nights, possibly using objective methods, would provide a more reliable picture of habitual sleep patterns and strengthen the interpretation of intervention outcomes. Additionally, we could not evaluate long-term effects of the intervention due to the limited follow-up period, and we do not know whether behavioural changes were sustained or not.

Our study also has several strengths, including the randomized controlled study design. The personalized design and the fact that the control group received the intervention at the three-month follow-up may have contributed to the high follow-up rate (88%). Additionally, the nature of the intervention inherently supports self-determination and goal setting^52,53^, both recognized as key components of effective behaviour change. Another strength is that the app was developed for both iOS and Android users, which in combination with the inclusion of both females and males, and participants with different levels of education, improve generalizability and broadens the applicability of our results.

## Conclusion

In conclusion, this three-month RCT evaluating a personalized app-based lifestyle intervention among working adults had small positive effects on exercise, , time in bed, sleep duration, and fruit and vegetable intake. The effect of the additional monthly coach support was unclear and should be evaluated in future studies. Although the effects were limited, even small improvements may be of clinical relevance given the dose-response relationships between these lifestyle factors and the risk for several noncommunicable diseases. In a broader perspective, personalized app-based interventions offer a scalable approach to support healthy lifestyle behaviours and have the potential to improve lifestyle behaviours, which may bring benefits both to individual and public health.

## Methods

### Study design

The Health Integrator study was a three-armed parallel RCT. We randomized participants 1:1:1 to a personalized app-based lifestyle intervention with app and an initial session with a health coach (app group), app with an initial session and additional monthly coach support (app-coach group), or to a control group. Inclusion criteria were: at least 18 years old, able to read and understand Swedish, access to and able to use a smartphone. We started recruitment and the baseline data collection in April 2018 and finished it in September 2018. The follow-up assessments were finalized in April 2019. These dates were in line with the study protocol^51^.

Details on the study design can be found elsewhere^51^. In summary, the study lasted for six months for each participant and comprised assessments at three time points: baseline, and three- and six-months follow-up. Participants were randomized by sex in blocks of six within each worksite, using a random allocation list generated by the author SEB using Stata. Each new participant was sequentially assigned to the next available position on the list at enrolment. Sample size was based on the primary outcome of health-related quality of life. Prior to study start, we calculated that 63 participants per group were sufficient to detect a difference of eight points (SD 16) in the general health domain of health-related quality of life questionnaire RAND-36^51^. This calculation was based on 80% power, a two-sided alpha level of .05 and the previously reported clinically meaningful difference^54^. Participants in the intervention groups received the three-month long intervention at baseline. To limit drop-out, participants in the control group received the intervention at the three-month follow-up. Therefore, we have only used data from baseline and the three-month follow-up to examine the effect of the intervention on lifestyle behaviours.

### Study participants

We recruited participants from four different worksites in Stockholm, Sweden. Two of the worksites were defined as white collar (office employees) and two as blue collar (bus drivers). The rationale for recruiting from the different worksites was to increase heterogeneity in characteristics, lifestyle behaviours, and environments. Based on communication with representatives at the Human Resources Departments at the different worksites, participants were recruited in two different ways. To the office employees, the Human Resources Department sent an inquiry email about the study. For the bus drivers, study personnel were present in person at the bus garages and those who were interested provided their private email addresses, since the employer had no record of email addresses. Convenience sampling was used and all employees at any worksite could participate if they fulfilled the inclusion criteria. Those interested in participating signed up for the study digitally and then downloaded the smartphone-app developed for the study, the Health Integrator app^33,51^. Through the app, they provided their informed consent to participate in the study and thereafter they provided their personal information, responded to a health profile, and filled out a research questionnaire. The health profile included questions on the six different lifestyle behaviours: physical activity, diet, alcohol, sleep, stress and smoking. The research questionnaire was approximately fifty pages (screens) with 2–10 items per page. Completeness was automatically checked. A unique identifier was attached to each email, thereby hindering participants from registering more than one response to each questionnaire.

After finalizing the questionnaire, the participants scheduled themselves for the baseline study meeting with a health coach, which took place at the participant’s worksite. Two health coaches were involved in the study. Each participant was assigned to the same coach throughout all time points. The participants were consecutively allocated and evenly distributed between the two coaches across worksites and study groups. At the baseline meeting, the health coach collected a written informed consent, performed anthropometric measurements and then informed the participants about their group allocation. Due to the nature of the study, participants and study personnel were unblinded to the group allocations. The health profile, the research questionnaire and the anthropometric measurements were repeated at the three-month follow-up.

Prior to the start of the study, a standardized strategy was established to ensure consistency in the conduct of study meetings and health coaching sessions. The coaching strategy was based on the Self-Determination Theory with the aim to encourage curiosity and initial motivation while supporting participants’ sense of competence and autonomy^55^. Participants were guided to formulate SMART goals, i.e. specific, measurable, action-oriented, realistic, time-bound, and accepted (self-determined)^56^.

### Intervention

**Fig. 2:**
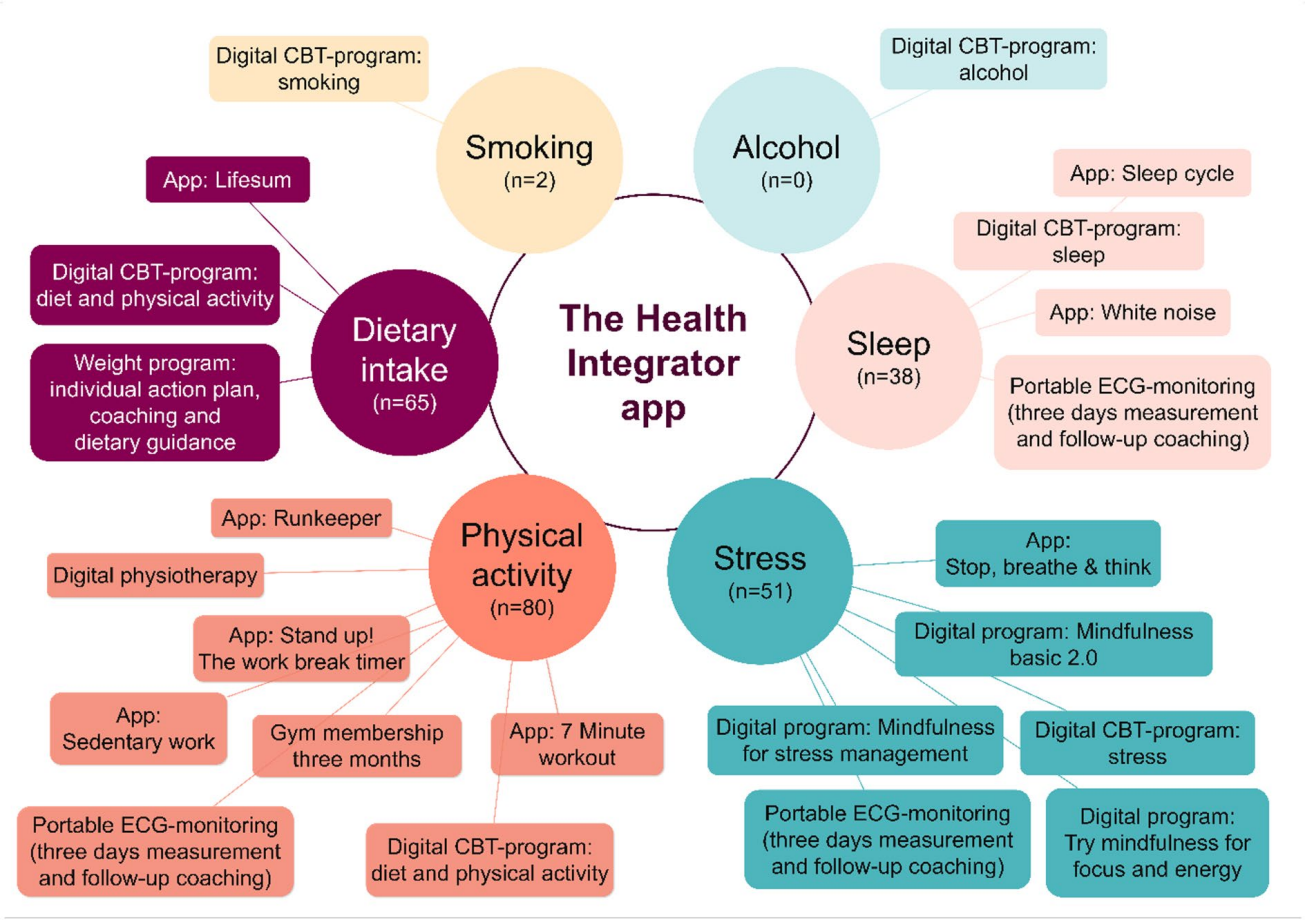
Available services in the Health Integrator study, a three-month randomized controlled trial, performed among working adults in Sweden. The numbers in parentheses represent how many participants that targeted each specific behaviour, among those included in analyses (*n* = 180). Participants could choose one or more services and services targeting different behaviours. CBT= Cognitive Behavioural Therapy, ECG= Electrocardiogram.

In addition to the assessments at the baseline meeting, participants in the two intervention groups reviewed a color-coded summary of the personal health profile. Based on this, the participant and the coach jointly identified one or more lifestyle behaviours to target in the next three months. Thereafter, in the Health Integrator app, a new feature was activated for participants assigned to the intervention groups. They were now able to choose from intervention services targeting physical activity, diet, alcohol, sleep, stress and smoking, for example a gym membership, a 12-week diet and physical activity program, or a stress-reducing mindfulness program, Fig. 2. Participants could choose one or more services targeting different behaviours and did not have to pay for the app nor the selected services. The intervention exclusively targeted lifestyle behavioural change and was primarily based on Social Cognitive Theory, with a particular emphasis on self‑efficacy and self‑regulation^57^. In addition, the intervention incorporated elements consistent with Self‑Determination Theory, most notably the emphasis on autonomy through the personalized design of the intervention^55^. Further, depending on the selected intervention services, specific behaviour‑change techniques were utilized, including goal setting, self‑monitoring, and reinforcement^58^.

In addition to the services, participants in the app-coach group also received sessions via telephone with their health coach twice (after one and after two months) during the intervention period. These coaching sessions aimed to build on what was discussed at baseline, including the participant’s goals and the use of the chosen services, as well as to discuss if any adjustments to the plan, or the goals, were needed. Session durations were approximately 20–30 min.

Participants in the app group and the control group did not receive any coaching support.

### Characteristics

Participants’ height and weight were measured and used to calculate BMI (kg/m^2^). Sex, education level, type of work, and date of birth were self-reported in the questionnaire. Age was calculated using date of birth and date of study meeting.

### Outcomes

#### Physical activity

In the research questionnaire, participants reported their weekly minutes of everyday activity (e.g. walking, biking, gardening) and exercise (e.g. running, gymnastics, ball sports) using two questions developed by the Swedish National Board of Health and Welfare and validated for clinical practice^59,60^. For everyday activity, response alternatives were: no time, < 30 min, 30–60 min, 60–90 min, 90–150 min, 150–300 min and > 300 min (5 h). For exercise, response alternatives were: almost never, < 30 min, 30–60 min, 60–90 min, 90–120 min and > 120 min. Responses were converted to numeric variables using the mean value of intervals or the specified numbers for the lowest and highest categories. We also created a combined variable of total physical activity by summarizing the two questions. For data analysis, everyday activity (min/week), exercise (min/week) and total physical activity (min/week) were retrieved.

For baseline characteristics, the participants’ physical activity levels were compared against the weekly recommendation of 150 min of moderate intensity or 75 min of vigorous intensity^35^. Everyday activity was added to the doubled minutes of exercise, and each participant was defined as either reaching the recommendation or not^35^.

#### Diet

Diet was assessed in the research questionnaire using a validated 92-item Food Frequency Questionnaire (FFQ)^61^. Portion sizes were either predefined in the FFQ or estimated using standard portion sizes. To calculate average daily energy intake, we utilized the Swedish National Food Agency’s food database^62^. For data analysis, energy intake (kilocalorie [kcal]/day), fruit (g/day), vegetables (g/day) and fruit and vegetables combined (g/day) were retrieved.

#### Alcohol intake

The FFQ in the research questionnaire also included questions about frequency and amount of intake for low-alcohol beer, medium-strength beer, strong beer, white wine, red wine, dessert wine and liquor. The frequency and amount of each item were used to summarize average intake, expressed as units per day, corresponding to number of standard units containing 12 g of alcohol. For data analysis, the average number of standard units of alcohol consumed (units/day) was retrieved.

#### Sleep

Selected questions from the Karolinska sleep questionnaire^63^ were used to assess sleep in the research questionnaire. Participants were asked to report the time they went to bed the previous night, how long it took them to fall asleep, and what time they woke up this morning. For data analysis, time in bed (h/night) and sleep duration (h/night) were retrieved.

#### Stress

We assessed stress in the research questionnaire using the Perceived Stress Scale^63,64^ which comprises fourteen items with two subscales: the negativity subscale (seven items) and the positivity subscale (seven items). Responses were coded from zero to four, with the positively stated questions reversely coded. The total scores range from 0 to 56, where a higher score indicates higher levels of perceived stress. For data analysis, stress (score) was retrieved.

#### Smoking

Current smoking habits were assessed in the health profile. The response options that started with “No” were followed by “I have never been a smoker”, “but I quit smoking more than 6 months ago” or “but I quit smoking less than 6 months ago”. The options that started with “Yes” were followed by “but not every day”, “I smoke 1–9 cigarettes per day”, “I smoke 10–19 cigarettes per day” or “I smoke more than 19 cigarettes per day”. Never and previous smokers were categorized as non-smokers, while current smokers were categorized as smokers. For data analysis, current smoking status (smoker, non-smoker) was retrieved.

### Ethical considerations

The study was performed in accordance with the Declaration of Helsinki, approved by the Regional Ethical Review board in Stockholm, Sweden (2018/411 − 31 and 2018/1038-32), and registered at ClinicalTrials.gov on May 6, 2018 (NCT03579342). Participation in the study was voluntary, and all participants provided informed consent. All collected data was de-identified by replacing personal identifiers with a study ID number. The key linking these numbers to individual participants was stored separately from the other collected data. All results are reported on group level. Further, individual results were not disclosed to the respective worksites, ensuring participant confidentiality. No financial or other compensation was offered for participation. All collected data was stored at safe servers at Karolinska Institutet, Sweden.

### Statistical analyses

Descriptive statistics are presented using mean and SD for continuous variables and frequencies and percentages (%) for categorical variables, both for all participants and separately for each study group. Analysis of variance and chi-squared tests were used to compare baseline characteristics between the groups. The two intervention groups were compared regarding the distribution of each target behaviour and the number of targeted behaviours using chi-squared tests.

For all analyses of the intervention effects, an intention-to-treat approach was applied. In other words, participants were consecutively evaluated based on their group allocation regardless of their adherence to the intervention. We had no access to adherence data on their chosen intervention services, i.e. we do not know to what extent participants, for example, utilized their gym membership or performed mindfulness exercises.

For the main analyses, the intervention effect on the continuous outcomes were examined using robust linear regression models, since we had two time points for assessments^65^ and to minimize the influence of outliers among the residuals. The difference between baseline and follow-up for each outcome was calculated and used as the dependent variable in linear regression models. The differences were examined in relation to group allocation using the control group as reference and models were adjusted for baseline values of each outcome, as recommended by Twisk et al.^65^. Smoking was the only categorical outcome, however, due to few smokers at baseline (*n* = 13) we could not analyse the intervention effect.

To examine any difference between participants who remained or dropped out in the study, we performed Little’s missing completely at random test and observed statistical significance (*p*<.001). Further, we compared baseline characteristics between those who remained or dropped out using independent t-test (age) and chi-squared tests (sex and education level). The difference was neither statistically significant for age (*p* = .90), nor for education level (*p*=.07), however, a higher proportion of males (*n* = 21/24) than females (*n* = 3/24) dropped out (*p*=.006, φ = 0.19). Therefore, to account for missing data at follow-up, we conducted two types of sensitivity analyses on the main outcomes and included all participants with available baseline data (*n* = 204). First, the last observation carried forward method was performed, imputing the baseline value to the follow-up variable. Second, we imputed the missing outcome variables using multiple imputation via chained equations, with the baseline values of each variable, sex, age and education level as input variables, and generated 20 imputations for each missing value.

In additional analyses, we stratified participants within each intervention group based on whether the specific lifestyle behaviour was targeted with a service in the Health Integrator app or not. For example, analyses of the intervention effect on physical activity were stratified by if physical activity was targeted with a service or not. Three participants did not choose any service in the app, but had instead manually written their targeted behaviours in the app (physical activity *n* = 3, diet *n* = 2, stress *n* = 1, sleep *n* = 1). This information was used to define their target behaviours. Four participants only focused on improvement of body weight (*n* = 1, received a scale) or blood pressure (*n* = 3, received a blood pressure monitor). In all the stratified analyses, these four participants were included in the group that did not target the certain lifestyle behaviour. At the three-month follow-up, two participants did not finish the entire questionnaire and did not respond to the FFQ, the perceived stress scale or the sleep questionnaire. One participant had reported an implausible energy intake, i.e. more than three standard deviations away from the sex-specific mean of the log-transformed energy intake. In addition, six participants had misinterpreted the sleep question and reported that they fell asleep before they went to bed. These participants were excluded from analyses of diet, stress and sleep, respectively. Besides these errors, we had no missing data on item level. We did not perform stratified analyses on alcohol, since no one chose the intervention service targeting alcohol. The stratified analyses were conducted post hoc and should be interpreted as exploratory.

*P*-values were two-tailed and *p*-values < .05 were considered statistically significant. Statistical analyses were performed using Stata 17.0 (Stata Corp).

## Supplementary Information

Below is the link to the electronic supplementary material.

Supplementary Material 1

## Data Availability

The datasets generated and analysed during this study are not publicly available due to ethical reasons, but are available from the corresponding author on reasonable request.
