# Supplementary material for: Effects on secondary outcomes following a three-month personalized app-based lifestyle intervention among working adults: a three-armed randomized controlled trial
Source: Sci Rep. 2026 May 31;16:16811. doi: 10.1038/s41598-026-54919-w (PMC13226666; doi:10.1038/s41598-026-54919-w)
Supplement: Supplementary file 1 — Supplementary Material 1 [file 41598_2026_54919_MOESM1_ESM.pdf]

Supplementary Tables

Supplemental Table S1 – Baseline characteristics of all participants with baseline data in the Health Integrator study, a three-month randomized controlled trial, performed among working adults in Sweden.

	All (n=204) mean (SD)	Control (n=66) mean (SD)	App (n=70) mean (SD)	App-coach (n=68) mean (SD)	*p* [a]
Age, years	48.3 (10.0)	48.5 (8.7)	47.7 (10.5)	48.6 (10.8)	.83
BMI, kg/m^2	27.1 (4.6)	27.1 (4.6)	26.7 (4.7)	27.5 (4.7)	.59
Physical activity, min/week					
Everyday activity	182.5 (106.6)	172.0 (108.1)	198.2 (102.0)	176.5 (109.4)	.31
Exercise	57.3 (47.9)	62.0 (49.1)	54.4 (49.5)	55.6 (45.5)	.61
Total physical activity	239.8 (136.7)	234.1 (144.6)	252.6 (129.4)	232.1 (137.1)	.62
Diet, intake/day [b]					
Energy, kcal	2162 (919)	2274 (1113)	2125 (897)	2091 (714)	.47
Fruit, g	199.9 (179.8)	197.1 (221.2)	196.9 (151.9)	205.6 (163.5)	.95
Vegetables, g	158.6 (126.2)	144.4 (159.3)	154.0 (91.2)	177.1 (120.3)	.30
Fruit and vegetables, g	358.5 (270.9)	341.5 (355.5)	350.9 (200.1)	382.7 (240.1)	.65
Alcohol, units/day [b]	0.8 (1.1)	0.9 (1.3)	0.8 (1.0)	0.6 (0.9)	.21
Sleep, h/night					
Time in bed	7.1 (1.3)	7.0 (1.4)	7.2 (1.2)	7.2 (1.2)	.66
Sleep duration	6.7 (1.3)	6.6 (1.4)	6.8 (1.3)	6.8 (1.2)	.61
Stress, score [c]	22.9 (7.6)	23.7 (8.5)	22.2 (7.1)	22.9 (7.1)	.51
	n (%)	n (%)	n (%)	n (%)	*p* [e]
Sex					.92
Females	78 (38.2)	24 (36.4)	27 (38.6)	27 (39.7)	
Males	126 (61.8)	42 (63.6)	43 (61.4)	41 (60.3)	
Education level					.78
≤12 years	101 (49.5)	32 (48.5)	37 (52.9)	32 (47.1)	
>12 years	103 (50.5)	34 (51.5)	33 (47.1)	36 (52.9)	
Type of work					.87
Office employee	98 (48.0)	33 (50.0)	34 (48.6)	31 (45.6)	
Bus driver	106 (52.0)	33 (50.0)	36 (51.4)	37 (54.4)	
Smoking status					.11
Smoker	16 (7.9)	9 (13.6)	4 (5.9)	3 (4.4)	
Non-smoker	186 (92.1)	57 (86.4)	64 (94.1)	65 (95.6)	
Physical activity, ≥150 min/week					.48
Yes	150 (73.5)	46 (69.7)	55 (78.6)	49 (72.1)	
No	54 (26.5)	20 (30.3)	15 (21.4)	19 (27.9)	
Target behaviour [e]					
Physical activity	87 (63.0)	-	43 (61.4)	44 (64.7)	.69
Diet	73 (52.9)	-	39 (55.7)	34 (50.0)	.50
Alcohol	0 (0.0)	-	0 (0.0)	0 (0.0)	-
Sleep	41 (29.7)	-	19 (27.1)	22 (32.4)	.50
Stress	57 (41.3)	-	25 (35.7)	32 (47.1)	.18
Smoking	2 (1.4)	-	2 (2.9)	0 (0.0)	.16

BMI = Body Mass Index, kcal = kilocalorie, [a] analysis of variance, [b] 92-item Food Frequency Questionnaire, [c] Perceived Stress Scale, [d] chi-squared tests, [e] possible to target one or more behaviours, only available in the intervention groups (n=138)

Supplemental Table S2 – Baseline and three-month follow-up data on lifestyle behaviours in the Health Integrator study, a three-month randomized controlled trial among working adults in Sweden. Behaviours are summarized by intervention group and further stratified by whether the behaviour was targeted or not.

	Control			App			App-coach		
	n	Baseline mean (SD)	Follow-up mean (SD)	n	Baseline mean (SD)	Follow-up mean (SD)	n	Baseline mean (SD)	Follow-up mean (SD)
**Entire group**									
Everyday activity, min/week [a]	60	172.3 (107.8)	172.5 (106.0)	57	204.5 (95.2)	192.9 (87.1)	63	186.9 (106.3)	186.4 (106.9)
Exercise, min/week [a]	60	65.0 (48.3)	60.0 (45.8)	57	53.7 (49.5)	69.2 (41.0)	63	58.8 (44.9)	70.2 (43.4)
Total physical activity, min/week [a]	60	237.3 (143.5)	232.5 (137.3)	57	258.2 (122.6)	262.1 (109.2)	63	245.7 (131.7)	256.7 (125.6)
Energy intake, kcal/day [b]	59	2258 (1134)	2072 (799)	55	2011 (714)	2075 (818)	63	2118 (711)	2097 (711)
Fruit, g/day [b]	59	209.6 (228.8)	188.7 (133.9)	55	194.0 (153.7)	196.1 (147.8)	63	215.2 (165.9)	223.0 (139.2)
Vegetables, g/day [b]	59	151.2 (166.5)	137.3 (95.0)	55	156.7 (94.7)	165.1 (100.8)	63	185.0 (121.1)	204.7 (167.9)
Fruit and vegetables, g/day [b]	59	360.8 (369.7)	326.0 (193.3)	55	350.7 (200.8)	361.3 (220.9)	63	400.2 (240.2)	427.7 (243.4)
Alcohol, units/day [b, d]	60	1.0 (1.3)	0.8 (1.3)	55	0.8 (0.9)	0.8 (0.9)	63	0.6 (0.9)	0.6 (0.6)
Time in bed, h/night [a]	59	7.0 (1.4)	7.0 (1.2)	56	7.2 (1.2)	7.3 (1.0)	57	7.3 (1.2)	7.5 (1.2)
Sleep duration, h/night [a]	59	6.6 (1.5)	6.7 (1.3)	56	6.9 (1.3)	6.8 (1.2)	57	6.9 (1.2)	7.2 (1.2)
Stress, score [c]	60	23.4 (8.5)	21.5 (8.8)	55	22.3 (7.6)	19.6 (7.4)	63	22.5 (7.1)	20.0 (6.3)
**Intervention** [e]									
Everyday activity, min/week [a]	-	-	-	37	206.8 (99.0)	192.6 (93.0)	43	185.9 (110.1)	194.3 (106.0)
Exercise, min/week [a]	-	-	-	37	46.2 (52.5)	70.5 (41.7)	43	52.3 (40.1)	68.7 (43.6)
Total physical activity, min/week [a]	-	-	-	37	253.0 (131.8)	263.1 (119.5)	43	238.3 (129.5)	263.0 (122.5)
Energy intake, kcal/day [b]	-	-	-	32	1900 (532)	1933 (734)	31	2157 (686)	2045 (707)
Fruit, g/day [b]	-	-	-	32	182.3 (114.7)	182.3 (95.3)	31	200.8 (156.2)	206.1 (112.8)
Vegetables, g/day [b]	-	-	-	32	145.0 (86.7)	164.8 (88.1)	31	175.8 (123.3)	190.0 (117.0)
Fruit and vegetables, g/day [b]	-	-	-	32	327.4 (141.1)	347.0 (129.8)	31	376.6 (244.7)	396.1 (191.8)
Time in bed, h/night [a]	-	-	-	17	7.1 (1.3)	7.5 (0.7)	19	7.3 (1.4)	7.5 (1.0)
Sleep duration, h/night [a]	-	-	-	17	6.7 (1.5)	7.2 (0.6)	19	6.8 (1.4)	7.1 (0.9)
Stress, score [c]	-	-	-	21	25.0 (8.8)	21.9 (9.2)	29	23.6 (7.0)	20.6 (6.9)
**No intervention** [f]									
Everyday activity, min/week [a]	-	-	-	20	200.3 (90.2)	193.5 (77.4)	20	189.0 (100.4)	169.5 (109.5)
Exercise, min/week [a]	-	-	-	20	67.5 (41.2)	66.8 (40.6)	20	72.8 (52.3)	73.5 (44.0)
Total physical activity, min/week [a]	-	-	-	20	267.8 (106.1)	260.3 (89.9)	20	261.8 (138.4)	243.0 (134.3)
Energy intake, kcal/day [b]	-	-	-	23	2165 (900)	2271 (901)	32	2080 (744)	2147 (723)
Fruit, g/day [b]	-	-	-	23	210.2 (197.4)	215.5 (200.4)	32	229.2 (176.1)	239.4 (160.8)
Vegetables, g/day [b]	-	-	-	23	172.9 (104.7)	165.7 (118.3)	32	193.9 (120.2)	219.0 (206.7)
Fruit and vegetables, g/day [b]	-	-	-	23	383.1 (262.7)	381.1 (308.8)	32	423.1 (237.5)	458.4 (284.5)
Time in bed, h/night [a]	-	-	-	39	7.3 (1.2)	7.3 (1.1)	38	7.2 (1.1)	7.5 (1.3)
Sleep duration, h/night [a]	-	-	-	39	7.0 (1.2)	6.7 (1.3)	38	6.9 (1.1)	7.2 (1.3)
Stress, score [c]	-	-	-	34	20.6 (6.3)	18.3 (5.8)	34	21.6 (7.1)	19.6 (5.9)

kcal = kilocalorie, [a] self-reported, [b] 92-item Food Frequency Questionnaire, [c] Perceived Stress Scale, [d] stratified analyses not performed since no participant targeted this behaviour, [e] *Intervention* includes the participants that targeted the certain behaviour, [f] *No intervention* includes the participants that did not target the certain behaviour

Supplemental Table S3 – Robust linear regression examining the difference in change between baseline and follow-up, stratified by if the behaviour was targeted or not, in comparison to the control group. Bold numbers imply statistical significance. Outcomes in the Health Integrator study, a three-month randomized controlled trial, performed among working adults in Sweden.

		Intervention [a]			No intervention [b]		
	Group	n	Difference [c] mean (SD)	Linear regression model [d] β (95% CI)	n	Difference [c] mean (SD)	Linear regression model [d] β (95% CI)
Everyday activity, min/week [e]	Control	60	0.3 (71.9)	ref	60	0.3 (71.9)	ref
	App	37	-14.2 (103.6)	-1.9 (-36.0, 32.2)	20	-6.8 (74.8)	3.9 (-28.9, 36.7)
	App-coach	43	8.4 (88.8)	13.1 (-16.9, 43.1)	20	-19.5 (138.9)	-13.2 (-69.1, 42.6)
Exercise, min/week [e]	Control	60	-5.0 (41.7)	ref	60	-5.0 (41.7)	ref
	App	37	24.3 (40.5)	**21.6 (8.1, 35.0)**	20	-0.8 (32.8)	5.3 (-11.1, 21.7)
	App-coach	43	16.4 (34.8)	**16.2 (2.8, 29.5)**	20	0.8 (40.9)	9.0 (-8.5, 26.5)
Total physical activity, min/week [e]	Control	60	-4.8 (89.2)	ref	60	-4.8 (89.2)	ref
	App	37	10.1 (120.0)	20.0 (-19.9, 60.0)	20	-7.5 (82.2)	8.5 (-29.1, 46.1)
	App-coach	43	24.8 (96.8)	29.8 (-4.0, 63.7)	20	-18.8 (168.4)	-5.0 (-71.7, 61.8)
Energy intake, kcal/day [f]	Control	59	-186 (1011)	ref	59	-186 (1011)	ref
	App	32	33 (655)	3 (-283, 289)	23	106 (952)	234 (-139, 608)
	App-coach	31	-112 (742)	13 (-273, 300)	32	67 (788)	142 (-145, 429)
Fruit, g/day [f]	Control	59	-20.9 (196.3)	ref	59	-20.9 (196.3)	ref
	App	32	-0.1 (102.9)	2.6 (-37.3, 42.5)	23	5.2 (164.2)	26.6 (-47.4, 100.6)
	App-coach	31	5.3 (137.9)	20.3 (-24.7, 65.3)	32	10.2 (220.7)	44.2 (-21.3, 109.7)
Vegetables, g/day [f]	Control	59	-13.8 (166.2)	ref	59	-13.8 (166.2)	ref
	App	32	19.7 (74.2)	29.2 (-5.7, 64.1)	23	-7.2 (99.4)	19.1 (-33.6, 71.7)
	App-coach	31	14.2 (112.2)	45.5 (-1.8, 92.9)	32	25.1 (147.1)	63.4 (-6.4, 133.2)
Fruit and vegetables, g/day [f]	Control	59	-34.8 (344.1)	ref	59	-34.8 (344.1)	ref
	App	32	19.7 (105.5)	30.3 (-26.2, 86.9)	23	-2.0 (216.6)	48.1 (-67.1, 163.3)
	App-coach	31	19.5 (195.5)	65.6 (-9.5, 140.8)	32	35.3 (313.6)	112.9 (-1.1, 226.9)
Time in bed, h/night [e]	Control	59	0.02 (1.25)	ref	59	0.02 (1.25)	ref
	App	17	0.44 (1.11)	**0.48 (0.08, 0.87)**	39	-0.05 (1.37)	0.14 (-0.29, 0.57)
	App-coach	19	0.18 (1.29)	0.38 (-0.08, 0.84)	38	0.29 (1.25)	0.44 (-0.01, 0.89)
Sleep duration, h/night [e]	Control	59	0.08 (1.27)	ref	59	0.08 (1.27)	ref
	App	17	0.52 (1.24)	**0.51 (0.12, 0.89)**	39	-0.29 (1.59)	-0.16 (-0.67, 0.34)
	App-coach	19	0.30 (1.52)	0.35 (-0.15, 0.85)	38	0.30 (1.15)	0.39 (-0.06, 0.84)
Stress, score [g]	Control	60	-1.9 (6.0)	ref	60	-1.9 (6.0)	ref
	App	21	-3.1 (4.9)	-0.9 (-3.4, 1.7)	34	-2.4 (5.2)	-1.4 (-3.6, 0.8)
	App-coach	29	-3.0 (5.7)	-1.0 (-3.5, 1.4)	34	-2.0 (6.7)	-0.7 (-3.1, 1.7)

kcal = kilocalorie, [a] *Intervention* includes the participants that targeted the certain behaviour, [b] *No intervention* includes the participants that did not target the certain behaviour, [c] difference baseline and follow-up, [d] adjusted for baseline value, [e] self-reported, [f] 92-item Food Frequency Questionnaire, [g] Perceived Stress Scale
